# Diagnostic ultrasound enhances, then reduces, exogenously induced brain activity of mice

**DOI:** 10.3389/fnhum.2024.1509432

**Published:** 2025-02-11

**Authors:** Henry Tan, Devon J. Griggs, Lucas Chen, Kahte Adele Culevski, Kathryn Floerchinger, Alissa Phutirat, Gabe Koh, Nels Schimek, Pierre D. Mourad

**Affiliations:** ^1^Department of Neurological Surgery, University of Washington, Seattle, WA, United States; ^2^Department of Electrical and Computer Engineering, University of Washington, Seattle, WA, United States; ^3^Washington National Primate Research Center, Seattle, WA, United States; ^4^Division of Engineering and Mathematics, University of Washington, Bothell, WA, United States

**Keywords:** diagnostic ultrasound, ultrasound, ultrasound stimulation, focused ultrasound, visual stimulation, neuromodulation, electrocorticography, LIFU

## Abstract

Transcranially delivered diagnostic ultrasound (tDUS) applied to the human brain can modulate those brains such that they became more receptive to external stimulation relative to sham ultrasound exposure. Here, we sought to directly measure the effect of tDUS on mouse brain activity subjected to an external stimulation—a blinking light. Using electrocorticography, we observed a substantial increase in median brain activity due to tDUS plus a blinking light relative to baseline and relative to sham tDUS plus a blinking light. Subsequent brain activity decreased after cessation of tDUS but with continuation of the blinking light, though it remained above that demonstrated by mice exposed to only a blinking light. In a separate experiment, we showed that tDUS alone, without a blinking light, had no observable effect on median brain activity, but upon its cessation, brain activity decreased. These results demonstrate that *simultaneous* exposure to tDUS and blinking light can increase the receptivity of the visual cortex of mice exposed to that light, and that *prior* exposure to tDUS can reduce subsequent brain activity. In each case, these results are consistent with published data. Our results on mice echo published human results but do not directly explain them, since their test subjects received less intense diagnostic ultrasound than did our mice. Given the near ubiquity of diagnostic ultrasound systems, further progress along this line of research could one day lead to the widespread use of *diagnostic* ultrasound to intentionally modulate human brain function during exogenous stimulation.

## 1 Introduction

### 1.1 Ultrasound background

Ultrasound has played important roles in medical care for many years (e.g., Soni and Lucas, 2015; Miller et al., 2012). Diagnostic ultrasound (DUS) illuminates a plane of tissue approximately a millimeter thick and at least as wide as the scan head itself (typically 2–3 cm). For those sufficiently trained, use of DUS can rapidly triage people with potential internal bleeding (the FAST exam – Quinn and Sinert, 2011), monitor fetal development (Kenneth, 1975), and other applications. Typical parameter ranges (Hoskins et al., 2019) include the carrier frequency of the ultrasound wave (~1–30 MHz), the length of pulses (1–3 pressure cycles for imaging, ~10–20 for Doppler analysis), number of pulses per second or pulse repetition frequency (PRF, ~1 kHz for imaging, ~5–10 kHz for Doppler), and intensity (less than 0.7 W/cm^2^ spatial peak, temporal average value (Ispta) as determined by the FDA). Brain imaging applications require carrier frequencies in the lower portion of that range, to facilitate transmission through human skull (~2 MHz) with all other parameters staying the same.

Recent therapeutic applications of ultrasound take advantage of the ability of devices to produce focused ultrasound (FUS) in small volumes (~10s of microliters) in which large physical effects can occur (heating, cavitation, tissue or fluid displacement), thereby producing therapeutic effects (Mourad, 2013; Izadifar et al., 2020). For example, high-intensity focused ultrasound (HIFU) delivered transcranially, can ablate brain tissue primarily through heating (Zhou, 2011; Krishna et al., 2023) and can break up tissue primarily via cavitation (Xu et al., 2021; Choi et al., 2024). Typical parameters include lower carrier frequencies (< 1 MHz), longer individual treatment pulses (seconds to minutes) and much greater intensities (> 1 kW/cm^2^ Ispta) relative to DUS. FUS at lower intensities and in conjunction with acoustic microbubbles can enhance drug delivery into the brain (Burgess et al., 2015; Mainprize et al., 2019; Bunevicius et al., 2020).

Transcranially-delivered focused ultrasound (tFUS) for modulating (e.g., activating or suppressing) focal brain function has gained increasing attention in recent years, as reviewed in and appreciated by comparing Fini and Tyler (2017); Bobola et al. (2018); Blackmore et al. (2019); Spivak et al. (2022) and Murphy and Fouragnan (2024).

Compared to DUS, typical tFUS parameters (e.g., Blackmore et al., 2019) include lower frequencies (~0.25–2.0 MHz), longer pulses (10s-100s of cycles), and higher intensities (0.5–5 W/cm^2^ Ispta), all of which are typically smaller than for HIFU. PRF values are typically either comparable or not applicable, since tFUS like HIFU application may be continuous.

### 1.2 Ultrasound can alter human brain’s response to external stimulation

Most research on ultrasound-facilitated neuromodulation study its effects in isolation, asking in essence: how does the application of tFUS to a particular portion of neuroanatomy change its intrinsic fuction? In contrast, Legon et al. (2014) showed that tFUS applied to human somatosensory cortex made that cortex more sensitive to external stimulation (two-point discrimination test; differential frequency vibration). Liu et al., 2021 demonstrated improved sensory discrimination caused by tFUS, due to its ability to excite the sensory cortex. Butler et al. (2022) showed that tFUS applied to the visual motion processing cortex of healthy human subjects improved their ability to track seen objects.

Importantly, little is known about the effects of transcranial diagnostic ultrasound (tDUS) on brain function compared to tFUS, given the typically higher carrier frequency and reduced intensity of tDUS relative to tFUS. However, there exist two reports on this subject. Gibson et al. (2018) showed that human motor cortex subjected to 2 min of tDUS responded to transcranial magnetic stimulation at lower intensity levels than those exposed to sham ultrasound. Schimek et al. (2020) demonstrated that repeated but intermittent application of tDUS toward the visual cortex induced illusory visual percepts in human participants as they stared at a mark on a computer screen.

Together, Gibson et al. (2018) and Schimek et al. (2020) motivate our hypothesis: that transcranially delivered diagnostic ultrasound can enhance the brain’s response to external stimuli.

Following Fry (1956) and Fry et al. (1958), who first demonstrated using a cat model the ability of ultrasound to alter the brain’s response to external light exposure, we measured brain activity in anesthetized mice subjected to an external stimulation — a (sham) blinking light — simultaneously with (sham) tDUS, then continued blinking light stimulation without ultrasound. We hypothesized that tDUS would enhance the brain’s response to external stimulation by light relative to light exposure alone. We further hypothesized that tDUS alone would not activate brain. Our experiments support these hypotheses. In addition, however, we observed that the enhanced activity due to tDUS plus light persisted after discontinuing tDUS, activity that remained above levels associated with continued blinking light exposure alone. These results are consistent with recent literature on the persistence of ultrasound’s modulation of brain function. Finally, we observed for those mice exposed to only tDUS, that their brain activity decreased after subsequent cessation of tDUS exposure: an unexpected finding about which we offer informed speculation.

## 2 Materials and methods

### 2.1 Approval

All animal procedures were vetted and approved by the University of Washington’s Institutional Animal Care and Use Committee under protocol number 4084–10.

### 2.2 Surgery

**Figure 1:**
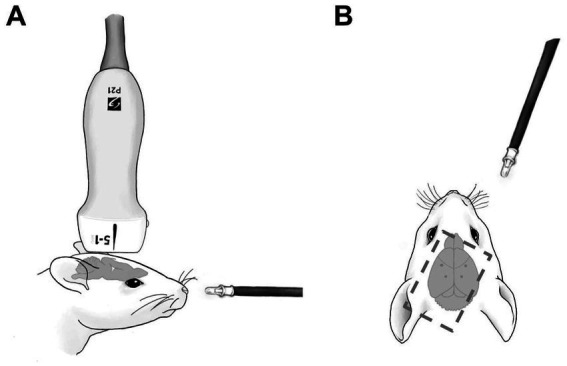
Schematic of experimental setup. **(A)** Shows setup from the side. **(B)** Shows setup from above. Dotted line shows the position and orientation of the ultrasound scan head. Dots in the mouse brain of **(B)** denote the position of ECoG electrodes.

Fourteen C57BL/6 mice (Jackson Laboratories, Farmington CT) were randomly assigned into two cohorts (*n* = 7), one to receive periodic blinking light stimulation with tDUS (the ‘US+Light’ cohort), the other to receive periodic blinking light stimulation without tDUS (the ‘Light-only’ cohort). A subsequent cohort of mice received tDUS without light stimulation (*n* = 6; the ‘US-only’ cohort). These animal numbers are consistent with those used by others in the field (e.g., Baek et al., 2020; Xu et al., 2023; Zhao et al., 2023). After induction of an adequate anesthetic plane with isoflurane gas, verified via toe pinch, each mouse was placed in a mouse stereotax (WPI, Sarasota, FL) with their heads held in place via ear bars and a bite bar. A light coat of artificial tears (Akorn, Lake Forest, IL) was then applied to both eyes to prevent corneal drying for the duration of the experiment. Subsequently, fur was removed from the scalp via 1 min application of Nair. Subcutaneous injection of lidocaine (2.0 mg/kg) and bupivacaine (2 mg/kg) (AuroMedics, East Windsor, NJ) was given as a local anesthetic. Initial vertical midline incision along the dorsal aspect of the scalp was then achieved using a no. 10 scalpel, exposing the underlying skull and anatomical landmarks. 25G x 5/8″ needles were then used to hand-drill holes in the skull in which to implant four in-house made electrocorticography (ECoG) electrodes (Pinnacle Technology, 8,415), one inserted into each of the visual (AP = -3.5 mm, ML = ±2.2 mm, DV = 1.0 mm) and somatosensory cortices (AP = -0.2 mm, ML = ±2.5 mm, DV = 2.0 mm), to brain cortex, with depths determined by a mouse brain atlas. We used a four-channel ECoG array (LabChart, ADInstruments, Inc., Colorado Springs, CO) to sample brain activity at 20 kS/s. To minimize interference between the delivery of ultrasound and the ECoG wires, we bent the wires away from the site of insertion, the DU transducer, and from the sides of the head. After placement of the electrodes and application of approximately 10 mm^3^ of Aquasonics ultrasound gel (Parker, Clinton Township, MI), the mouse and stereotaxic were enclosed within a custom-built copper Faraday cage to shield the mouse from the electromagnetic pulses generated by the ultrasound device while still allowing ultrasound transmission into mouse brain. More ultrasound gel was placed on the cage and over the mouse to ensure adequate transcranial ultrasound transmission into the brain. A micropositioner and a series of clamps and vice grips were used to hold the ultrasound scan head as it was lowered into place over the left visual and right somatosensory cortices, such that the center of the transducer was directly above the left visual cortex (AP = -3.5 mm, ML = -2.2 mm) – Figure 1.

### 2.3 Experimental design

#### 2.3.1 Exposure to light

After the procedures listed above, an LED light driven by a function generator (Agilent Technologies, Santa Clara, CA) was placed approximately 2–3 centimeters from the right eye of the mouse. We then made a five-minute baseline brain-activity recording with the light turned off. Next, for the two cohorts of mice that we exposed to a blinking light (0.1 s in duration, followed by 9.9 s of background room light), we performed a light-titration experiment to obtain a light intensity value to use for the subsequent part of the experiment, specific to each mouse. Specifically, the voltage driving the light was manipulated within the range of 2.47–5 V to achieve a minimal response to the light from each mouse as assessed in real-time. 2.47 V was the lowest voltage at which the LED would generate observable light. These titration experiments were conducted to achieve approximately a 50% response rate and lasted 12.29 ± 5.03 min per mouse; the titration ensured the voltage level selected for optical stimulation of each mouse was neither too weak to generate any brain activity, nor too strong to always generate brain activity, which may have made the effects of tDUS difficult to observe. For mice exposed to only tDUS, prior to experimention, we kept the mice under anesthesia with ophthalmic cream and exposed them to only background room light for a length of time comparable to the average of that used to titrate the intensity of blinking light in the other experiments.

#### 2.3.2 Exposure to ultrasound

To deliver the ultrasound as described below, we used a P21x5–1 scan head deployed by a Sonosite MicroMaxx using the same device and settings as used by McClintic et al. (2014). Supplementary Table S1 lists the ultrasound parameters of this exposure. For the sham tDUS trials, we used the same scan head deployed in the same way, but without turning on the ultrasound.

#### 2.3.3 Experimental steps – exposure to light plus (sham) ultrasound

**Figure 2:**
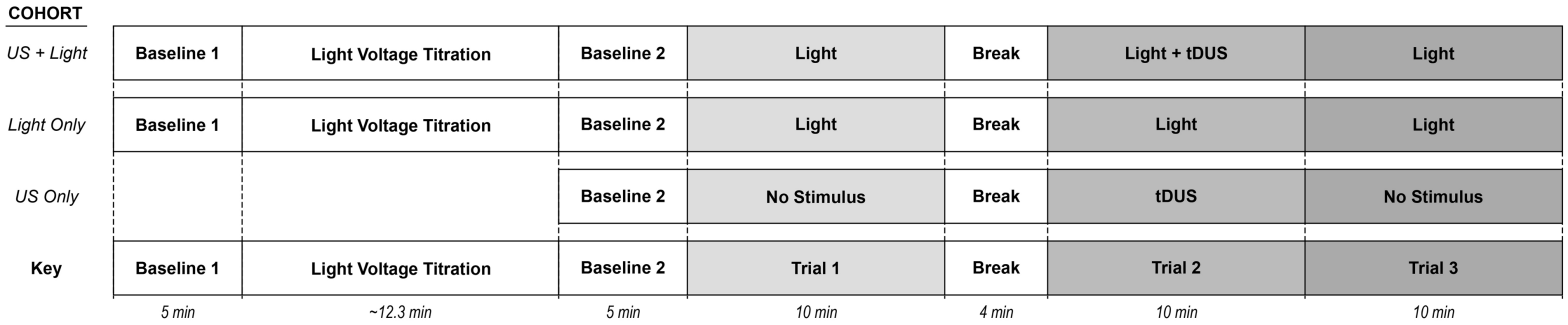
Schematic representation of the experiment for a given mouse. Here, Trial 1 refers to mice exposed only to (sham) blinking light. Trial 2 refers to mice exposed to (sham) blinking light plus (sham) tDUS. Trial 3 refers to mice exposed only to a (sham) blinking light. The Light+US cohort received each of blinking light and tDUS. The Light-only cohort experienced only a blinking light. The US-only cohort received only tDUS.

After the titration step, we performed three separate trials that together we define as one experiment as illustrated in Figure 2.

Regarding the first two trials, performed together, mice from the ‘Light-only’ and ‘US+Light’ cohorts were allowed to rest under anesthesia for a second five-minute baseline, after the titration step and before the experiment began. The RMS value of the last minute of this second baseline was used to normalize all our trial data. Then, to summarize, Trial 1 consisted of exposing each mouse to only a blinking light, Trial 2 consisted of exposing each mouse to a blinking light simultaneously with (sham) tDUS, and Trial 3 consisted of exposing each mouse to only a blinking light. In greater detail, Trial 1 consisted of exposing the right eye of each mouse to a blinking light of 0.1 s in duration, every 10 s, using the voltage determined during the titration portion of the experiment. We define each such 10 s interval as an “event.” Each mouse was exposed to approximately 60 events during Trial 1. We followed this first light exposure phase with a four-minute time period during which the mice remained under anesthesia without exposure to the blinking light. Trial 2 then followed, consisting of (sham) tDUS paired with the same light exposure paradigm as in Trial 1 (10 min of exposure to 0.1 s light stimulus every 10 s, for approximately 60 events). Trial 2 was then followed immediately by Trial 3 (10 min of exposure to 0.1 s light stimulus every 10 s, for approximately 60 events).

As a control, we performed a separate experiment to test whether tDUS alone could activate brain. This separate cohort of mice (the ‘US-only’ cohort) underwent the three Trial protocols outlined above without turning on the blinking light before or during any of the three Trials, hence exposing them to only background light throughout the experiment. Instead, they experienced tDUS during only the second trial. We analyzed these control results against those derived from the Light-only and US+Light cohorts described above.

### 2.4 ECoG data collection and initial data conditioning

**Figure 3:**
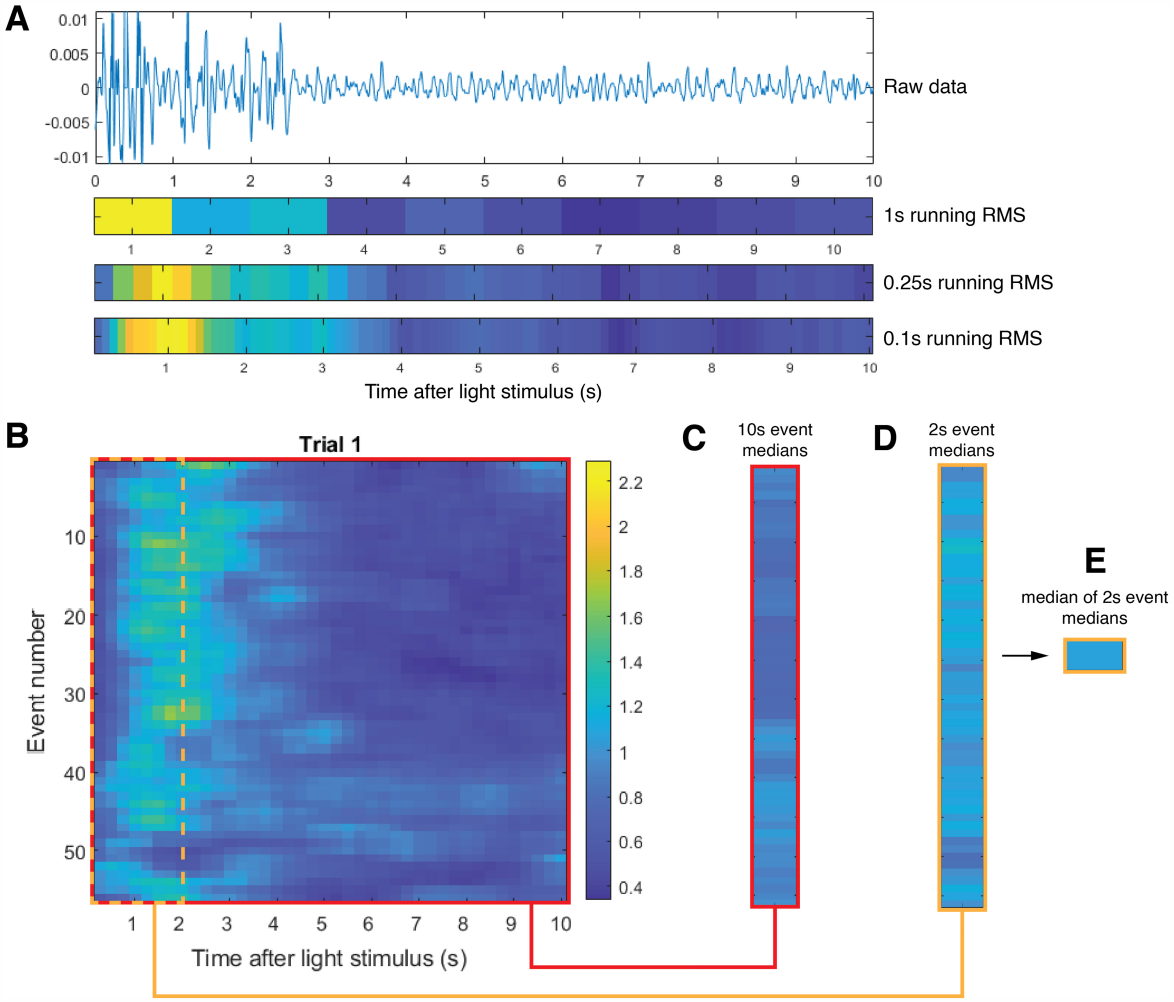
Schematic of analysis, here applied to sample representative data. **(A)** Sample baseline-normalized ECoG data from one light event (from top to bottom): raw data, 1 second block RMS (tick marks denote center of time segment), 0.25-second running RMS (used in our analysis) and 0.1-second running RMS (tick marks denote end of time segment). **(B)** Representative set of 55 brain-activity events collected during the entire Trial 1 for a single mouse. Each row contains 0.25-second running RMS brain-activity data. The red box outlines the entire set of brain activity events for this mouse. The dotted green box outlines the first two seconds of that data. **(C)** Results of averaging the 0.25-second running RMS of each event over the entire ten seconds for each event. **(D)** Results of averaging the 0.25-second running RMS of each event over the first two seconds of each event. **(E)** Calculation of the median of all 55 calculations shown in **(D)**. We used this median value calculated for each mouse to normalize all the event data for each mouse in all Trials.

LabChart (ADInstruments, Inc., Colorado Springs, CO) was used to sample brain activity via our ECoG arrays at 20 kS/s. MATLAB (Mathworks, Natick, MA) was used to analyze the ECoG signals, first by applying a zero-phase 5–55 Hz filter with MATLAB’s *butter* and *filtfilt* functions. Data with RMS values greater than four standard deviations were removed from analysis (Krugliak and Clarke, 2022). Initial analysis of the data showed that light-induced brain activity could span up to 4 s after its initiation. We therefore divided our data into much smaller segments — choosing 0.25 s increments — producing a running RMS time series for each light event using MATLAB’s *movmean* function over all of the light events for each trial type for each mouse (Figure 3). Pooling these data for each trial type across all mice produced ~60 values per mouse and ~420 values per cohort for a given trial type.

### 2.5 ECoG data analysis

We initially normalized the RMS time series of our ECoG data for each 10 s (light) event within every trial by each animal’s median RMS value of cortical activity as measured during the last minute of their second baseline session, after titration but before the start of Trial 1 (Figure 3). Subsequent statistical analysis showed meaningful differences in brain activity between the Light-only versus Light+US cohorts during Trial 1 for subsets of the events, such as comparing two cohorts after the first second of every event. When comparing activity among the three cohorts of mice, all subsets of events (1 through 10 s) demonstrated a statistically significant difference. To account for these differences in brain activity during Trial 1, we chose to normalize all the trial data with Trial 1 data, for each mouse. Specifically, we first calculated the RMS value of median brain activity of the data from all ~60 events for a given mouse from Trial 1. We then used that RMS value in subsequent analysis to normalize that mouse’s Trial 1, Trial 2 and Trial 3 data. For example, when analyzing whether the brain activity of the cohorts were different from one another 3 s into an event between Trial 2 and Trial 3, we would use the RMS value calculated for each mouse across all ~60 events in Trial 1, up to 3 s of each event, to normalize the RMS data for all ~60 events in Trial 2 and Trial 3, up to 3 s of each event.

We used ratio normalization to avoid generating bias, which can occur with other normalization methods such as z-scoring (Ciuparu and Mureşan, 2016). For each mouse Trial data set, we normalized that data by the *average* of that mouse’s Trial 1 data, to avoid generation of bias that can occur when *each* trial is normalized to data immediately before the trial (Ciuparu and Mureşan, 2016). Further, we used wideband analysis (5–55 Hz) to avoid biases that can arise when analyzing ratios of narrow frequency bands (Donoghue et al., 2020), such as variation in center frequencies, which can lead to misleading estimations of band powers (Lansbergen et al., 2011).

### 2.6 Statistical analysis

We used Kruskal-Wallis analysis in MATLAB (Mathworks, Natick, MA) to determine whether there exists a statistically significant difference between the median values of three or more pairs of data, with a Bonferroni correction. Where appropriate, we applied Mann–Whitney U-Tests to determine whether or not pairs of data had statistically significant differences in their median value, with a Bonferroni correction. In each case, we specify the number of data sets and associated effective p-value—denoted as α—in our description of the results.

### 2.7 Resources

ECoG data were preprocessed and exported using a custom Python script designed for the batch conversion of *mat* files into the BrainVision file format (*vhdr*, *vmrk*, *eeg*) using the *MNE-Python* library for electrophysiological data handling to ensure compatibility with standard neuroimaging formats. Metadata were extracted and stored in *JSON* file format, detailing experimental parameters, recording conditions, and preprocessing steps. The dataset was organized according to the Brain Imaging Data Structure (BIDS) standard (Pernet et al., 2019), with subject and session-specific folders, and a *participants.tsv* file summarizing experimental subjects.

The complete dataset is openly available on OpenNeuro (https://doi.org/10.18112/openneuro.ds005688.v1.0.1), enabling reproducibility and further analysis by the scientific community. We have made our MATLAB code available to those interested on https://github.com/cIiche/vis_stim, repository ID: 659440878.

## 3 Results

### 3.1 Overview of the data

**Figure 4:**
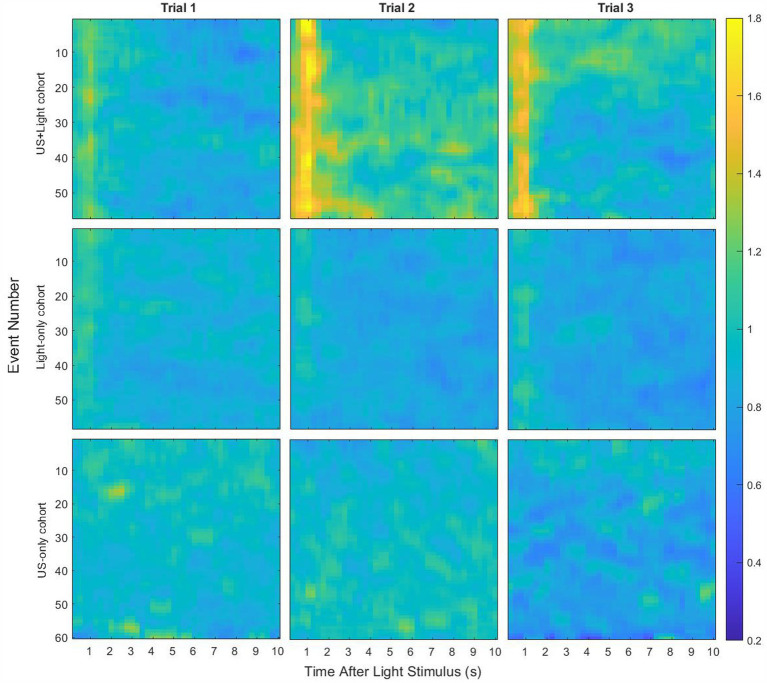
Median RMS brain activity in the brains of mice as a function of their cohort and Trial types, for event lengths of 10 s. Each line shows the median of a 0.25 s running RMS of brain activity during each event, normalized by baseline brain activity. Light stimulation, lasting 0.1 s, occurred at the start of each event.

Figure 4 shows the average median brain activity per cohort across all cohorts and trials. While missing the variance of this data, which we use below, we drew initial guidance from these results. We first noted that the blinking light generated a large value of RMS brain activity during the first few seconds of each event for the Light-only and US+Light cohorts, activity that was missing from the US-only cohort, whose mice were not exposed to blinking light. Next, we observed that the average data showed clear differences between the cohorts during Trial 1, before exposure of any of the mice to (sham) tDUS.

### 3.2 Short data segments showed the strongest influence of blinking light

**Figure 5:**
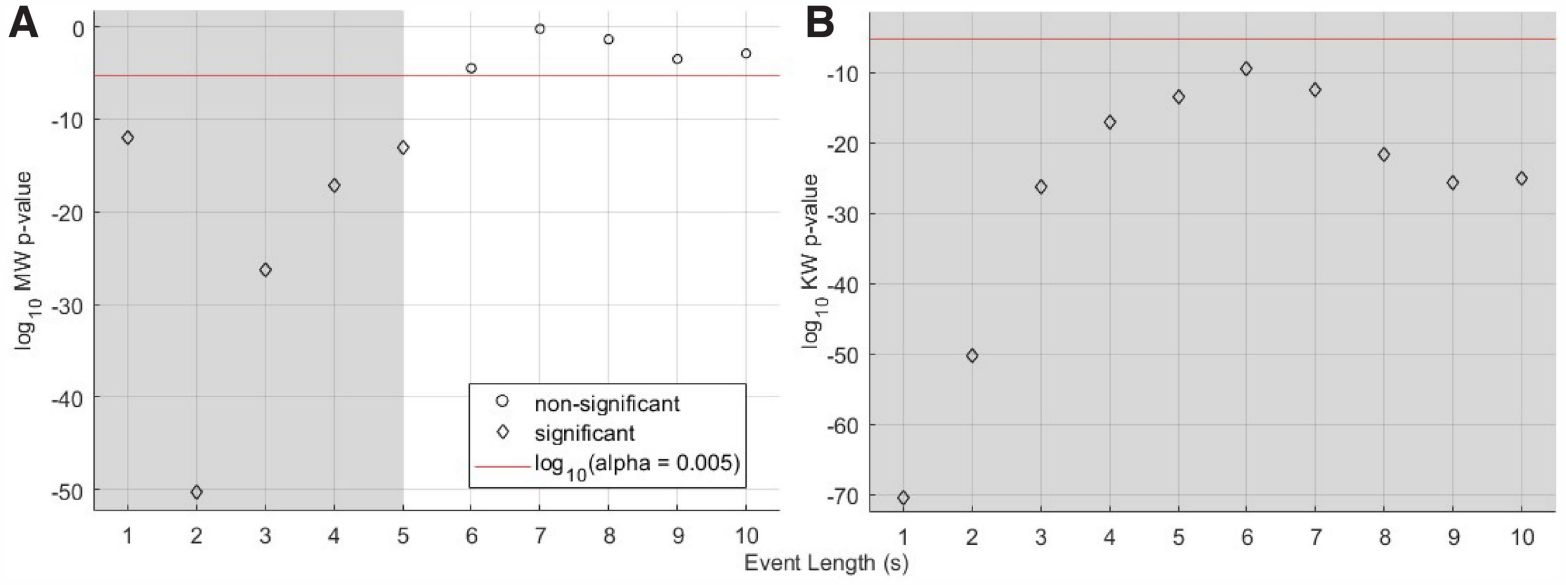
Statistical significance versus length of subset of events for Trial 1 cohorts, data normalized by baseline brain activity. **(A)** Mann-Whitney analysis p-value versus event length of median RMS brain activity for the US+Light and Light-only cohorts during the first Trial. **(B)** Kruskal-Wallis analysis of the US+Light, Light-only, and US-only cohorts. The red line denotes the log_10_ of the effective p-value (α = 0.005, adjusted for multiple comparisons of *n* = 10 pairs of data) below which values denote statistical significance between cohorts and above which comparisons do not differ in a statistically significant fashion. Grey areas denote statistically significant cases as a function of subset of event length.

These observations motivated our first analysis step: determination as a function of subsets of the 10 s event length if there exists a statistically significant difference in brain activity during Trial 1, when we expected that at least the Light-only and US+Light cohorts would have comparable brain activity. For those two cohorts, our analysis demonstrated a statistically significant difference in brain activity for subsets of event lengths (with durations of one through 5 s, inclusive) but not for longer subsets of event lengths (6 through 10 s event length)—Figure 5A. When we included all three cohorts from Trial 1, all subsets of event lengths demonstrated a statistically significant difference in brain activity during Trial 1—Figure 5B. These differences likely arose because of the different titration history of each mouse in each of the US+Light and Light-only cohorts, and the lack of exposure to light of the US-only cohort.

### 3.3 Trial 1 results differed between cohorts, requiring re-normalization of the data

We therefore normalized all subsets of event data for each mouse collected during the three Trials with the median RMS value of brain activity across all subsets of events from Trial 1, for each mouse. We then determined ranges of subsets of event lengths over which our normalized data demonstrated different characteristics. Kruskal-Wallis analysis showed that there exist statistically significant differences for each cohort across all three trials (α = 0.0017, *n* = 30 multiple comparisons, see Supplementary Table S2) and within each of Trials 2 and 3 for all three cohorts (α = 0.0025, *n* = 20 multiple comparisons, see Supplementary Table S3).

### 3.4 Intra-cohort statistical analysis

#### 3.4.1 Trial 1 versus trial 2 – determining significance versus length of event analyzed

**Figure 6:**
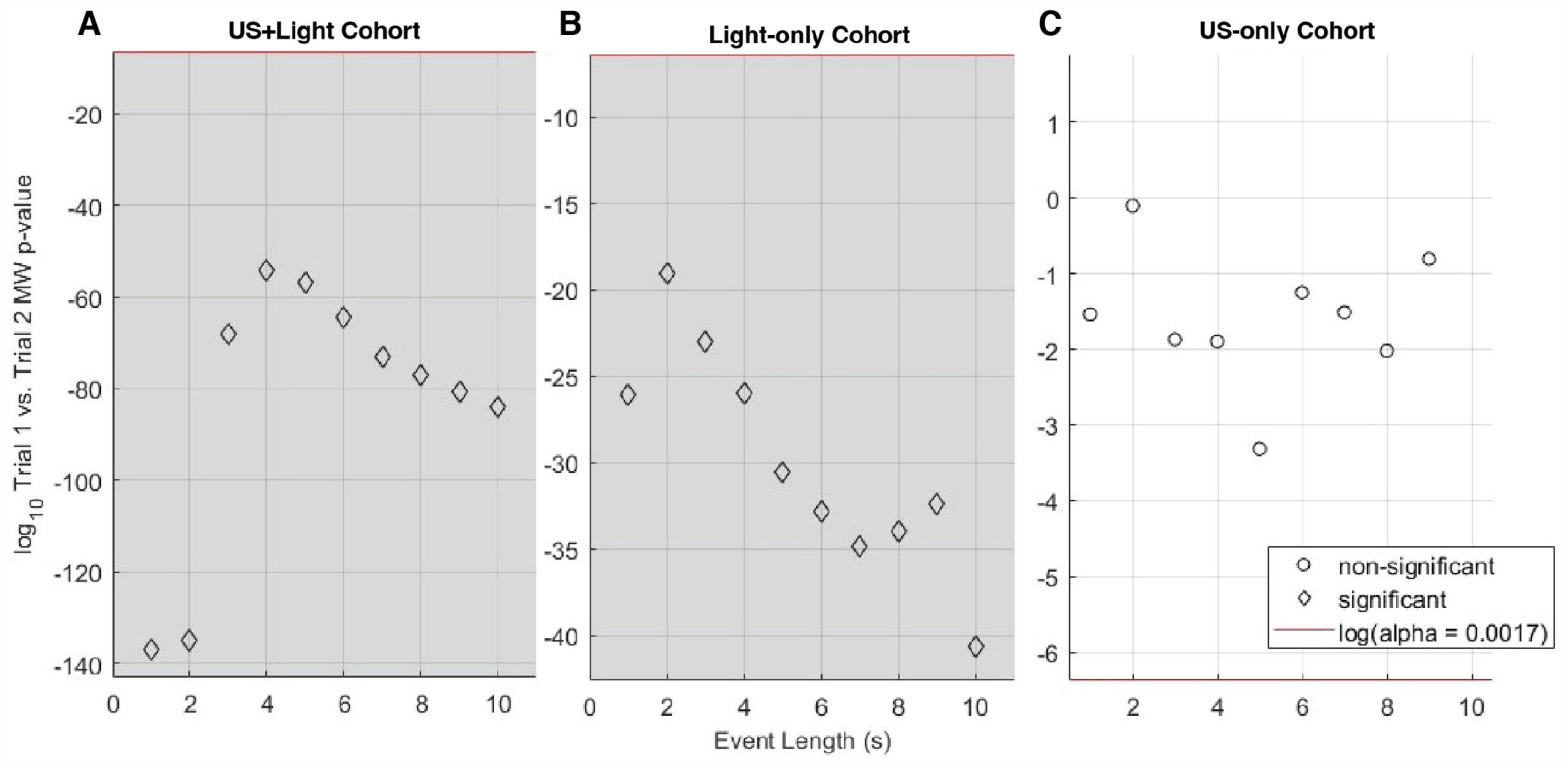
Log_10_ of the p-value for Mann-Whitney analysis of intra-cohort brain activity observed during Trial 1 versus Trial 2, as a function of subset of event length. **(A)** Shows this comparison within the US+Light cohort. **(B)** Shows this comparison within the Light-only cohort. **(C)** Shows this comparison within the US-only cohort. The red line denotes the log_10_ of the effective p-value (α = 0.0017, adjusted for multiple comparisons of *n* = 30 pairs of data) below which values denote statistical significance between cohorts and above which comparisons do not differ in a statistically significant fashion. Grey areas denote statistically significant cases as a function of subset of event length.

As a function of the length of event analyzed, the median RMS brain-activity values of the US+Light cohort always showed a difference in brain activity during Trial 2 versus Trial 1. This is when we applied tDUS plus blinking light, relative to its prior exposure to only a blinking light during Trial 1 (Figure 6A). The Light-only cohort also always showed a difference in brain activity during Trial 2 versus Trial 1 (Figure 6B). The US-only cohort showed no difference in brain activity during Trial 2 (tDUS exposure only) versus Trial 1 (no tDUS exposure, no blinking light exposure) – Figure 6C.

#### 3.4.2 Trial 2 versus trial 3 – determining significance versus data length analyzed

**Figure 7:**
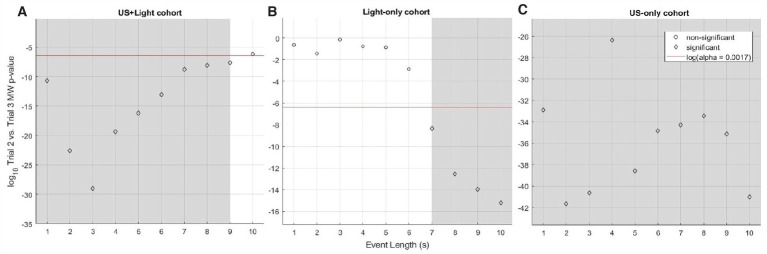
Log_10_ of p-value for Mann-Whitney analysis of intra-cohort brain activity observed during Trial 2 versus Trial 3 as a function of event length. **(A)** Shows this comparison within the US+Light cohort. **(B)** Shows this comparison within the Light-only cohort. **(C)** Shows this comparison within the US-only cohort. The red line denotes the log_10_ of the effective p-value (α = 0.0017, adjusted for multiple comparisons of *N* = 30 pairs of data) below which values denote statistical significance between cohorts and above which comparisons do not differ in a statistically significant fashion. Grey areas denote statistically significant cases as a function of subset of event length.

As a function of the length of event analyzed, the median RMS brain-activity values of the US+Light and Light-only cohorts dichotomized between short-and long-subsets of event lengths when comparing brain activity during Trial 2 versus Trial 3 (Figures 7A, B, respectively). In contrast, the brain activity of the US-only cohort brain always reduced during Trial 3 (no tDUS exposure) relative to Trial 2 (tDUS exposure) – Figure 7C.

### 3.5 Comparison of intra-cohort brain activity

**Figure 8:**
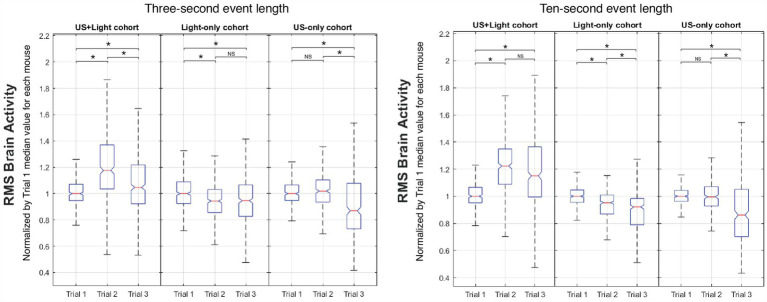
Representative intra-cohort comparison of brain activity versus Trial number. Meaningful differences occur as a function of event length between Trial 2 and Trial 3 for the US+Light and Light-only cohorts. NS denotes insignificant difference, while asterisk denotes statistical significance with *p* < 0.00056, adjusted for *n* = 90 multiple comparisons using Mann-Whitney. The central mark of each boxplot denotes the median value of the distribution of the data while the bottom and top of the box mark the 25th and 75th percentiles of the data, respectively. Whiskers enclose greater than 99% of the data.

Figure 8 shows representative intra-cohort brain activity plots versus Trial number with a short subset of event length (3 s) and with a long subset of event length (the entire 10-s event duration).

#### 3.5.1 Regarding the US+light cohort

It showed enhanced brain activity during Trial 2 (tDUS + blinking light) relative to that observed during Trial 1 (blinking light only, before tDUS delivery), consistent with our governing hypothesis. In addition, brain activity decreased during Trial 3 (blinking light; no tDUS) relative to that observed for Trial 2 for short subsets of event lengths, for which the majority of the data shows brain activity generated by the blinking light. Brain activity maintained (statistically speaking) during Trial 3 relative to that observed for Trial 2 for long subsets of event lengths, for which the majority of the data does not show brain activity generated by the blinking light—only background light from the room. In all cases, brain activity during Trial 3 for this cohort was above that measured during their first exposure to blinking light (Trial 1).

#### 3.5.2 Regarding the light-only cohort

It showed a small but statistically significant reduction in brain activity between Trials 1 and 2, independent of the subset of event length that we analyzed. The results for this cohort differed as a function of the subset of event lengths between Trial 2 and Trial 3: brain activity maintained for short subsets of event lengths, while it continued to decrease for long subsets of event lengths. This net decrease in brain activity exposed to blinking light during our experiment is consistent with the literature (Minamisawa et al., 2017).

#### 3.5.3 Regarding the US-only cohort

It showed no statistically significant change in brain activity between Trial 1 (before tDUS delivery) and Trial 2 (during tDUS delivery), also consistent with our hypothesis. In contrast, brain activity decreased during Trial 3 relative to Trial 1 and to Trial 2, when the mice experienced only background light from the room (no tDUS delivery). These results did not depend on the length of event data analyzed.

### 3.6 Inter-cohort analysis

**Figure 9:**
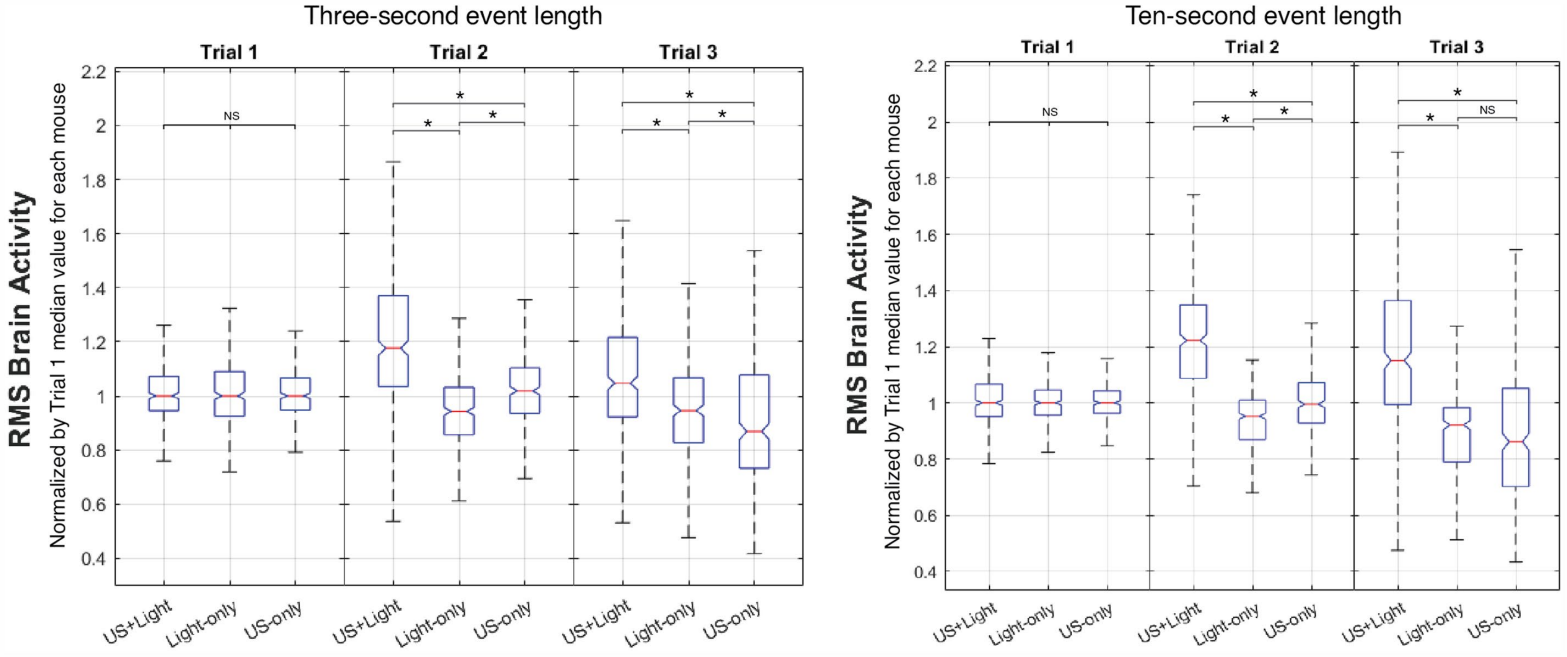
Representative intra-trial comparison of brain activity versus cohort type. Meaningful differences occur as a function of event length only during Trial 3. NS denotes insignificant difference, while asterisk denotes statistical significance, with *p* < 0.00083, adjusted for *n* = 60 multiple comparisons using Mann Whitney.

Figure 9 shows representative intra-trial brain activity plots versus cohort type with a short subset of event length (3 s) and with a long subset of event length (the entire 10-s event duration). Normalized RMS median brain activity for the US+Light group always had a greater value than that of the other groups for each of Trial 2 and Trial 3. During Trial 2, the US-only group always showed more normalized brain activity than did the Light-only group (though again it did not differ from brain activity during Trial 1). During Trial 3, brain activity in the US-only group reduced or trended toward reduction, depending on the subset of event length that we analyzed.

Finally, given the limited bandwidth of our data acquisition system, we could not resolve whether or not individual tDUS pulses generated individual brain activity events.

## 4 Discussion

We performed an experiment on three cohorts of mice (Figure 2). We exposed one cohort of mice (the ‘Light-only’ group) to a blinking light of 0.1 s in duration, otherwise background light in the room, every 10 s for our three Trials. We exposed a second cohort of mice (the ‘US-only’ group) to background light in the room (no blinking light) during the first 10-min Trial, then tDUS plus background light during the second 10-min Trial, then to only background light during the third 10-min Trial. We exposed a third cohort of mice (the ‘US+Light’ group) to blinking plus background light during the first Trial, then to blinking plus background light and tDUS during the second Trial, then to blinking plus background light during the third Trial.

We observed enhanced brain activity in the Light+US cohort when they experienced simultaneous tDUS and light relative to mice exposed to only a blinking and background light (Light-only cohort) and to mice exposed to only tDUS and background light (US-only cohort). These results are consistent with our governing hypothesis. The enhanced brain activity of the Light+US cohort persisted after cessation of tDUS (from Trial 2 to Trial 3). In contrast, brain activity for the US-only cohort did not change between 10 min of exposure to background light (Trial 1) then tDUS plus background light (Trial 2), though it did decrease upon cessation of tDUS but not background light (Trial 3).

Different aspects of our data are consistent with the observations of others, each with subtle differences in method and parameters that can yield robustly different results. Fry (1956) and Fry et al. (1958) reported early observations of a transient effect of ultrasound on brain function; they exposed an anesthetized cat first to a blinking light, then to (uncharacterized) ultrasound without a blinking light, then to more blinking light. They observed reversibly reduced amplitude of various components of the visually evoked potential (VEP) generated by the light after cessation of ultrasound. Similarly, Yoo et al. (2011) demonstrated that tFUS could either reduce or enhance various components of the VEP of anesthetized rabbits (measured with electrophysiological recordings and with fMRI) when applied simultaneously with a blinking light after prior exposure to a blinking light, depending on a range of specific tFUS parameters. Importantly, they also reported that delivery of tFUS without blinking light did not induce measurable brain activity. Also following Fry (1956), Kim et al. (2015) demonstrated in a rat model that simultaneous exposure of tFUS and blinking light in a dark room after prior exposure to only blinking light in a dark room could either enhance, reduce, or fail to change associated brain activity, depending again on a range of specific tFUS parameters.

Furthermore, we note that several papers demonstrate that the effect of tFUS on brain activity can persist beyond its time of application. For example, Fry (1956) and Fry et al. (1958) showed continued suppression of visually evoked potentials in a cat model lasting 30 min after cessation of ultrasound delivery. Yoo et al. (2011) showed that when observed, the reduction in brain activity persisted for approximately 10 min beyond the time of tFUS application. Yoo et al. (2018) applied tFUS to anesthetized rats subjected to electrical stimulation of their hind leg. Relative to sham application of tFUS, these authors observed differences in the shape of the observed sensory evoked potentials for up to 35 min after cessation of 10 min of tDUS. Dallapiazza et al. (2018) reported a reduction in the amplitude of somatosensory evoked potentials for at least 10 min after 40 s of tFUS to the thalamus of pigs. Folloni et al. (2019) reported discernible changes in endogenous activity between different neuroanatomical structures with the brains of macaques that lasted up to an hour after cessation of 40 s of tFUS. Niu et al. (2022) demonstrated that 5 min of tFUS applied to rat hippocampus induced observable changes in the structure of postsynaptic action potentials induced via direct electrical stimulation pre-synaptically, changes that lasted 45–60 min after cessation of ultrasound.

Regarding the results we report here, our observation of enhanced brain activity during simultaneous delivery of tDUS plus light (blinking and background) after exposure to just a blinking plus background light is consistent with some of the observations of Kim et al. (2015) and Yoo et al. (2011). Subsequent maintenance of enhanced brain activity after cessation of tDUS while in the presence of blinking plus background light is consistent with the other observations we cited above. Our US-only cohort, which experienced tDUS without blinking light (but with background light) during Trial 2, did not demonstrate increased brain activity during the time of application of tDUS, consistent with Yoo et al. (2011).

Motivated by the literature summarized above, we hypothesize that the observed subsequent reduction in brain activity for the US-only cohort during Trial 3 arose because tDUS during Trial 2 made the brains of these mice less receptive to *background* light during Trial 3 relative to the receptivity of their brains to background light during Trial 1, before exposure to tDUS. These observations are similar to those of Yoo et al. (2011), who used tFUS and a blinking light. We also hypothesize that the partial reduction in brain activity experienced by the US+Light cohort during Trial 3 occurred in part due to this same effect.

We also observed that tDUS with light produced stronger brain activity than light alone and that tDUS alone did not generate brain activity. We infer, therefore, that our tDUS dosage amounts to sub-threshold neural stimulation, i.e., stimulation too weak to evoke neural activity directly, but strong enough to enhance the neural responses generated by other stimuli. In our case, we observed that tDUS enhanced the brain’s response to signals originating from the eye. There exist observations of externally applied sub-threshold electrical energy altering CNS function, which motivates our inference. For example, sub-threshold electrical stimulation of the spine during therapeutic load bearing improved motor performance in rats with spinal cord injuries (Gad et al., 2013) and improved stroke rehabilitation in humans (Kim et al., 2021). Kim et al. (2021) hypothesized that electrical energy reduced the post-synaptic energy required for exogenous activation. Our results suggest that sub-threshold ultrasound stimulation is another way to create the same effect, and non-invasively, hence the underlying neural mechanisms deserve comparable exploration.

While motivated by Gibson et al. (2018) and Schimek et al., our work can only obliquely address their results. There exist meaningful differences between the exposure of transcranial diagnostic ultrasound to mice described here versus its transcranial exposure to humans, due to the significant differences in skull thickness between mice and humans. Those differences—significantly greater intensity and dose exposure for mice—require mitigation before mouse-based research can definitively inform the human condition.

### 4.1 Future work

We calculated the RMS of our ECoG data to highlight gross changes in brain function to test our primary hypotheses. Future research might consider search for frequency-dependent changes in brain function created by tDUS. tFUS, for example, delivered with a given PRF can generate activity at that frequency, e.g., enhanced gamma activity (Bobola et al., 2020; Park et al., 2021). Diagnostic ultrasound systems use PRF values that range from 1 to 10 kHz (Szabo, 2014). Relevant here, Dong et al. (2023) showed modulation of low-frequency activity in the theta band created by tFUS applied *in vivo* with a PRF of 1 kHz. Another line of research relies on the fact that neurosurgeons often use diagnostic ultrasound imaging intra-operatively to assess tumor margins that may have shifted between preoperative imaging and post-craniotomy (Mahboob et al., 2016). However, intra-operative brain stimulation to identify eloquent brain (Berger et al., 1989), when used with DUS (Silbergeld, 1994), may unintentionally influence the brain’s response to that stimulation. These observations suggest research of naming tasks during awake neurosurgery: those tasks may improve during and immediately after application of sufficient diagnostic ultrasound to Broca’s area and to Brodmann areas 44 and 45.

Active research built on advanced engineering concepts are steadily bringing modulation of brain function by tFUS forward toward eventual common use and analysis (Martin et al., 2024; Murphy and Fouragnan, 2024). In contrast, FDA-approved diagnostic ultrasound systems are nearly ubiquitous throughout the health-care system, world-wide. There exists, however little exploration of the potential use of DUS for neuromodulation, due to the intrinsic barriers for its reliable and sufficient transcranial delivery. Given the ready availability of such systems, our work and that of Gibson et al. and Schimek et al., along with future research that surmounts those barriers, may point the way toward eventual adoption of diagnostic ultrasound as a means of modulating the function of human brains subjected to exogenous stimulation.

## Data Availability

The complete dataset is available at OpenNeuro (Doi: https://doi.org/10.18112/openneuro.ds005688.v1.0.1), enabling reproducibility and further analysis by the scientific community. The MATLAB code available to those interested at https://github.com/ cIiche/vis_stim, repository ID: 659440878.

## References

[ref1] Baek H. Sariev A. Lee S. Dong S. Y. Royer S. Kim H. (2020). Deep cerebellar low-intensity focused ultrasound stimulation restores interhemispheric balance after ischemic stroke in mice. IEEE Trans. Neural Syst. Rehabil. Eng. 28, 2073–2079. doi: 10.1109/TNSRE.2020.3002207, PMID: 32746292

[ref2] Berger M. S. Kincaid J. Ojemann G. A. Lettich E. (1989). Brain mapping techniques to maximize resection, safety, and seizure control in children with brain tumors. Neurosurgery 25, 786–792. doi: 10.1227/00006123-198911000-00015, PMID: 2586730

[ref3] Blackmore J. Shrivastava S. Sallet J. Butler C. Cleveland R. (2019). Ultrasound neuromodulation: a review of results, mechanisms and safety. Ultrasound Med. Biol. 45, 1509–1536. doi: 10.1016/j.ultrasmedbio.2018.12.015, PMID: 31109842 PMC6996285

[ref4] Bobola M. Chen L. Ezeokeke C. Kuznetsova K. Lahti A. Lou W.. (2018). A review of recent advances in ultrasound, placed in the context of pain diagnosis and treatment. Curr. Pain Headache Rep. 22:60. doi: 10.1007/s11916-018-0711-7, PMID: 29987680 PMC6061208

[ref5] Bobola M. S. Chen L. Ezeokeke C. K. Olmstead T. A. Nguyen C. Sahota A.. (2020). Transcranial focused ultrasound, pulsed at 40 Hz, activates microglia acutely and reduces Aβ load chronically, as demonstrated in vivo. Brain Stimul. 13, 1014–1023. doi: 10.1016/j.brs.2020.03.016, PMID: 32388044 PMC7308193

[ref6] Bunevicius A. McDannold N. J. Golby A. J. (2020). Focused ultrasound strategies for brain tumor therapy. Oper. Neurosurg. (Hagerstown) 19, 9–18. doi: 10.1093/ons/opz374, PMID: 31853548 PMC7293897

[ref7] Burgess A. Shah K. Hough O. Hynynen K. (2015). Focused ultrasound-mediated drug delivery through the blood-brain barrier. Expert. Rev. Neurother. 15, 477–491. doi: 10.1586/14737175.2015.1028369, PMID: 25936845 PMC4702264

[ref8] Butler C. Rhodes E. Blackmore J. Cheng X. Peach R. Veldsman M.. (2022). Transcranial ultrasound stimulation to human middle temporal complex improves visual motion detection and modulates electrophysiological responses. Brain Stimul. 15, 1236–1245. doi: 10.1016/j.brs.2022.08.022, PMID: 36067978

[ref9] Choi S. W. Komaiha M. Choi D. Lu N. Gerhardson T. I. Fox A.. (2024). Neuronavigation-guided transcranial histotripsy (NaviTH) system. Ultrasound Med. Biol. 50, 1155–1166. doi: 10.1016/j.ultrasmedbio.2024.04.001, PMID: 38789304 PMC11822949

[ref10] Ciuparu A. Mureşan R. C. (2016). Sources of bias in single-trial normalization procedures. Eur. J. Neurosci. 43, 861–869. doi: 10.1111/ejn.13179, PMID: 26797876

[ref11] Dallapiazza R. Timbie K. Holmberg S. Gatesman J. Lopes M. Price R.. (2018). Noninvasive neuromodulation and thalamic mapping with low-intensity focused ultrasound. J. Neurosurg. 128, 875–884. doi: 10.3171/2016.11.JNS16976, PMID: 28430035 PMC7032074

[ref12] Dong S. Xie Z. Yuan Y. (2023). Transcranial ultrasound stimulation modulates neural activities during NREM and REM depending on the stimulation phase of slow oscillations and theta waves in the hippocampus. Cereb. Cortex 33, 8956–8966. doi: 10.1093/cercor/bhad174, PMID: 37222461

[ref13] Donoghue T. Dominguez J. Voytek B. (2020). Electrophysiological frequency band ratio measures conflate periodic and aperiodic neural activity. eNeuro 7:192. doi: 10.1523/ENEURO.0192-20.2020, PMID: 32978216 PMC7768281

[ref14] Fini M. Tyler W. (2017). Transcranial focused ultrasound: a new tool for non-invasive neuromodulation. Int. Rev. Psychiatry 29, 168–177. doi: 10.1080/09540261.2017.1302924, PMID: 28430535

[ref15] Folloni D. Verhagen L. Mars R. Fouragnan E. Constans C. Aubry J.. (2019). Manipulation of subcortical and deep cortical activity in the primate brain using transcranial focused ultrasound stimulation. Neuron 101, 1109–1116.e5. doi: 10.1016/j.neuron.2019.01.019, PMID: 30765166 PMC6520498

[ref16] Fry W. J. (1956). Ultrasound in neurology. Neurology 6, 693–704. doi: 10.1212/wnl.6.10.693, PMID: 13369651

[ref17] Fry F. Ades H. Fry W. (1958). Production of reversible changes in the central nervous system by ultrasound. Science 127, 83–84. doi: 10.1126/science.127.3289.83, PMID: 13495483

[ref18] Gad P. Choe J. Shah P. Garcia-Alias G. Rath M. Gerasimenko Y.. (2013). Sub-threshold spinal cord stimulation facilitates spontaneous motor activity in spinal rats. J. Neuroeng. Rehabil. 10:108. doi: 10.1186/1743-0003-10-108, PMID: 24156340 PMC4016220

[ref19] Gibson B. Sanguinetti J. Badran B. Yu A. Klein E. Abbott C.. (2018). Increased excitability induced in the primary motor cortex by transcranial ultrasound stimulation. Front. Neurol. 9:1007. doi: 10.3389/fneur.2018.01007, PMID: 30546342 PMC6280333

[ref170] Hoskins P. Martin K. Thrush A. (2019). Diagnostic ultrasound (CRC Press), 3rd edn., PMID: 13495483

[ref20] Izadifar Z. Izadifar Z. Chapman D. Babyn P. (2020). An introduction to high intensity focused ultrasound: systematic review on principles, devices, and clinical applications. J. Clin. Med. 9:460. doi: 10.3390/jcm9020460 32046072 PMC7073974

[ref21] Kenneth R. (1975). Gottesfeld, ultrasound in obstetrics and gynecology. Semin. Roentgenol. 10, 305–313. doi: 10.1016/0037-198x(75)90051-6, PMID: 1103298

[ref22] Kim H. Park M. Lee S. Lee W. Chiu A. Yoo S. (2015). Suppression of EEG visual-evoked potentials in rats through neuromodulatory focused ultrasound. Neuroreport 26, 211–215. doi: 10.1097/WNR.0000000000000330, PMID: 25646585 PMC4326564

[ref23] Kim K. Yoo S. J. Kim S. Y. Lee T. Lim S. H. Jang J. E.. (2021). Subthreshold electrical stimulation as a low power electrical treatment for stroke rehabilitation. Sci. Rep. 11:14048. doi: 10.1038/s41598-021-93354-x, PMID: 34234199 PMC8263745

[ref24] Krishna V. Fishman P. S. Eisenberg H. M. Kaplitt M. Baltuch G. Chang J. W.. (2023). Trial of Globus pallidus focused ultrasound ablation in Parkinson's disease. N. Engl. J. Med. 388, 683–693. doi: 10.1056/NEJMoa2202721, PMID: 36812432

[ref25] Krugliak A. Clarke A. (2022). Towards real-world neuroscience using mobile eeg and augmented reality. Sci. Rep. 12:2291. doi: 10.1038/s41598-022-06296-3, PMID: 35145166 PMC8831466

[ref26] Lansbergen M. M. Arns M. van Dongen-Boomsma M. Spronk D. Buitelaar J. K. (2011). The increase in theta/beta ratio on resting-state EEG in boys with attention-deficit/hyperactivity disorder is mediated by slow alpha peak frequency. Prog. Neuro-Psychopharmacol. Biol. Psychiatry 35, 47–52. doi: 10.1016/j.pnpbp.2010.08.004, PMID: 20713113

[ref27] Legon W. Sato T. Opitz A. Mueller J. Barbour A. Williams A.. (2014). Transcranial focused ultrasound modulates the activity of primary somatosensory cortex in humans. Nat. Neurosci. 17, 322–329. doi: 10.1038/nn.3620, PMID: 24413698

[ref28] Liu C. Yu K. Niu X. He B. (2021). Transcranial focused ultrasound enhances sensory discrimination capability through somatosensory cortical excitation. Ultrasound Med. Biol. 47, 1356–1366. doi: 10.1016/j.ultrasmedbio.2021.01.025, PMID: 33622622 PMC8011531

[ref29] Mahboob S. McPhillips R. Qiu Z. Jiang Y. Meggs C. Schiavone G.. (2016). Intraoperative ultrasound-guided resection of gliomas: a meta-analysis and review of the literature. World Neurosurg. 92, 255–263. doi: 10.1016/j.wneu.2016.05.007, PMID: 27178235

[ref30] Mainprize T. Lipsman N. Huang Y. Meng Y. Bethune A. Ironside S.. (2019). Blood-brain barrier opening in primary brain tumors with non-invasive MR-guided focused ultrasound: a clinical safety and feasibility study. Sci. Rep. 9:321. doi: 10.1038/s41598-018-36340-0, PMID: 30674905 PMC6344541

[ref31] Martin E. Aubry J. F. Schafer M. Verhagen L. Treeby B. Pauly K. B. (2024). ITRUSST consensus on tandardized reporting for transcranial ultrasound stimulation. Brain Stimul. 17, 607–615. doi: 10.1016/j.brs.2024.04.013, PMID: 38670224 PMC12436198

[ref32] McClintic A. King B. Webb S. Mourad P. (2014). Mice exposed to diagnostic ultrasound in utero are less social and more active in social situations relative to controls. Autism Res. 7, 295–304. doi: 10.1002/aur.1349, PMID: 24249575 PMC4025980

[ref33] Miller D. L. Smith N. B. Bailey M. R. Czarnota G. J. Hynynen K. Makin I. R. (2012). Bioeffects Committee of the American Institute of ultrasound in medicine. Overview of therapeutic ultrasound applications and safety considerations. J. Ultrasound Med. 31, 623–634. doi: 10.7863/jum.2012.31.4.623, PMID: 22441920 PMC3810427

[ref34] Minamisawa G. Funayama K. Matsumoto N. Matsuki N. Ikegaya Y. (2017). Flashing lights induce prolonged distortions in visual cortical responses and visual perception. eNeuro 4:2017:304. doi: 10.1523/ENEURO.0304-16.2017, PMID: 28508035 PMC5429040

[ref35] Mourad P. D. (2013). “Therapeutic ultrasound, with an emphasis on applications to the brain” in Ultrasonic transducers – Materials design and applications. eds. Nakamura K. Ueha S. (Cambridge: Woodhead Publishing Ltd.), 545–568.

[ref36] Murphy K. Fouragnan E. (2024). The future of transcranial ultrasound as a precision brain interface. PLoS Biol. 22:e3002884. doi: 10.1371/journal.pbio.3002884, PMID: 39471185 PMC11521279

[ref37] Niu X. Yu K. He B. (2022). Transcranial focused ultrasound induces sustained synaptic plasticity in rat hippocampus. Brain Stimul. 15, 352–359. doi: 10.1016/j.brs.2022.01.015, PMID: 35104664 PMC9295311

[ref38] Park M. Hoang G. M. Nguyen T. Lee E. Jung H. J. Choe Y.. (2021). Effects of transcranial ultrasound stimulation pulsed at 40 Hz on Aβ plaques and brain rhythms in 5×FAD mice. Transl. Neurodegener. 10:48. doi: 10.1186/s40035-021-00274-x, PMID: 34872618 PMC8650290

[ref39] Pernet C. R. Appelhoff S. Gorgolewski K. J. Flandin G. Phillips C. Delorme A.. (2019). Eeg-bids, an extension to the brain imaging data structure for electroencephalography. Sci. Data 6:103. doi: 10.1038/s41597-019-0104-8, PMID: 31239435 PMC6592877

[ref40] Quinn A. C. Sinert R. (2011). What is the utility of the focused assessment with sonography in trauma (FAST) exam in penetrating torso trauma? Injury 42, 482–487. doi: 10.1016/j.injury.2010.07.249, PMID: 20701908

[ref41] Schimek N. Burke-Conte Z. Abernethy J. Schimek M. Burke-Conte C. Bobola M.. (2020). Repeated application of transcranial diagnostic ultrasound towards the visual cortex induced illusory visual percepts in healthy participants. Front. Hum. Neurosci. 14:66. doi: 10.3389/fnhum.2020.00066, PMID: 32194387 PMC7062642

[ref42] Silbergeld D. L. (1994). Intraoperative transdural functional mapping. Technical note. J. Neurosurg. 80, 756–758. doi: 10.3171/jns.1994.80.4.0756, PMID: 8151360

[ref43] Soni N. J. Lucas B. P. (2015). Diagnostic point-of-care ultrasound for hospitalists. J. Hosp. Med. 10, 120–124. doi: 10.1002/jhm.2285, PMID: 25408226

[ref44] Spivak N. Tyler W. Bari A. Kuhn T. (2022). Ultrasound as a neurotherapeutic: a circuit-and system-based interrogation. Focus (Am Psychiatr Publ) 20, 32–35. doi: 10.1176/appi.focus.20210022, PMID: 35746933 PMC9063590

[ref45] Szabo T. L. (2014). Diagnostic ultrasound imaging: Inside out –. 2nd Edn. Amsterdam, Netherlands: Elsevier/Academic Press.

[ref46] Xu Z. Hall T. L. Vlaisavljevich E. Lee F. T. (2021). Histotripsy: the first noninvasive, non-ionizing, non-thermal ablation technique based on ultrasound. Int. J. Hyperth. 38, 561–575. doi: 10.1080/02656736.2021.1905189, PMID: 33827375 PMC9404673

[ref47] Xu R. S. Wu X. M. Xiong Z. Q. (2023). Low-intensity ultrasound directly modulates neural activity of the cerebellar cortex. Brain Stimul. 16, 918–926. doi: 10.1016/j.brs.2023.05.012, PMID: 37245844

[ref48] Yoo S. S. Bystritsky A. Lee J. H. Zhang Y. Fischer K. Min B. K.. (2011). Focused ultrasound modulates region-specific brain activity. NeuroImage 56, 1267–1275. doi: 10.1016/j.neuroimage.2011.02.058, PMID: 21354315 PMC3342684

[ref49] Yoo S. Yoon K. Croce P. Cammalleri A. Margolin R. Lee W. (2018). Focused ultrasound brain stimulation to anesthetized rats induces long-term changes in somatosensory evoked potentials. Int. J. Imaging Syst. Technol. 28, 106–112. doi: 10.1002/ima.22262, PMID: 29861548 PMC5975969

[ref50] Zhao Z. Ji H. Zhang C. Pei J. Zhang X. Yuan Y. (2023). (2023) modulation effects of low-intensity transcranial ultrasound stimulation on the neuronal firing activity and synaptic plasticity of mice. NeuroImage 270:119952. doi: 10.1016/j.neuroimage.2023.119952 36805093

[ref51] Zhou Y. F. (2011). High intensity focused ultrasound in clinical tumor ablation. World J. Clin. Oncol. 2, 8–27. doi: 10.5306/wjco.v2.i1.8, PMID: 21603311 PMC3095464

