# Supplementary material for: Diagnostic ultrasound enhances, then reduces, exogenously induced brain activity of mice
Source: Front Hum Neurosci. 2025 Feb 11;18:1509432. doi: 10.3389/fnhum.2024.1509432 (PMC11850526; doi:10.3389/fnhum.2024.1509432)
Supplement: Supplementary file 1 [file Data_Sheet_1.pdf]

Supplementary Material

1 SUPPLEMENTARY TABLES

Carrier Frequency	Cycles per pulse	Pulse Repetition Frequency	Duty cycle Duty cycle	Mechanical index	pressure pressure	Peak positive pressure	Spatial peak, temporal averaged intensity
2.0 MHz	3	3.5 kHz	0.8%	0.8	1.15 MPa	1.5MPa	0.62 ± 0.22 W/cm^2

Table S1. Diagnostic ultrasound parameters.

Time interval after the start of each event (s)	Kruskal-Wallis intercohort Trial 1	Kruskal-Wallis intercohort Trial 2	Mann Whitney between cohorts at Trial 2 (p)			Kruskal-Wallis intercohort Trial 3	Mann Whitney between cohorts Trial 3 (p)		
	p	p	US+Light vs. Light-only	US+Light vs. US-only	Light-only vs. US-only	p	US+Light vs. Light-only	US+Light vs. US-only	Light-only vs. US-only
1	0.146	*	*	*	*	*	*	*	0.0710
2	0.747	*	*	*	*	*	*	*	*
3	0.600	*	*	*	*	*	*	*	*
4	0.218	*	*	*	*	*	*	*	3.40e-3
5	0.323	*	*	*	*	*	*	*	*
6	0.644	*	*	*	*	*	*	*	0.0190
7	0.666	*	*	*	*	*	*	*	0.205
8	0.905	*	*	*	*	*	*	*	0.146
9	0.891	*	*	*	*	*	*	*	0.139
10	0.953	*	*	*	*	*	*	*	0.404

Table S2. Intracohort Kruskal Wallis and and Mann-Whitney test results on cohort-pooled median RMS values as a function of event length analysis, comparing trials within each cohort. Asterisks denote statistical significance for p<0.0025, adjusted for N=20 multiple comparisons.

Time interval analyzed after the start of each event (s)	Kruskal-Wallis intracohort US+Light cohort	Mann Whitney between trials US+Light cohort (p)			Kruskal-Wallis intracohort Light-only cohort	Mann Whitney between cohorts Light-only cohort (p)			Kruskal-Wallis intracohort US-only cohort	Mann Whitney between cohorts US-only cohort(p)		
	p	Trial 1 vs. Trial 2	Trial 2 vs. Trial 3	Trial 1 vs. Trial 3	p	Trial 1 vs. Trial 2	Trial 2 vs. Trial 3	Trial 1 vs. Trial 3	p	Trial 1 vs. Trial 2	Trial 2 vs. Trial 3	Trial 1 vs. Trial 3
1	*	*	*	*	*	*	0.569	*	*	0.214	*	*
2	*	*	*	*	*	*	0.243	*	*	0.903	*	*
3	*	*	*	*	*	*	0.911	*	*	0.680	*	*
4	*	*	*	*	*	*	0.686	*	*	0.151	*	*
5	*	*	*	*	*	*	0.595	*	*	0.0362	*	*
6	*	*	*	*	*	*	0.0796	*	*	0.281	*	*
7	*	*	*	*	*	*	*	*	*	0.218	*	*
8	*	*	*	*	*	*	*	*	*	0.130	*	*
9	*	*	*	*	*	*	*	*	*	0.440	*	*
10	*	*	2.15×10^{-3}	*	*	*	*	*	*	0.726	*	*

Table S3. Intercohort Kruskal Wallis and and Mann-Whitney test results on cohort-pooled median RMS values as a function of event length analysis, comparing trials between all three cohorts. Asterisks denote statistical significance for p<0.0017, adjusted for N=30 multiple comparisons.
